# Photon-counting CT *versus* energy-integrating detector and flat-panel CT for cadaveric wrist arthrography with additional tin filter dose reduction

**DOI:** 10.1186/s41747-025-00604-y

**Published:** 2025-08-29

**Authors:** Johannes de Boer, Nigar Salimova, Friederike Weidemann, Lea Behrendt, Thomas Werncke, Frank K. Wacker, Lena Sonnow

**Affiliations:** 1 Department of Diagnostic and Interventional Radiology, Hannover Medical School, Hannover, Germany; 2 Department of Trauma Surgery, Hannover Medical School, Hannover, Germany

**Keywords:** Arthrography, Cadaver, Musculoskeletal system, Tomography (x-ray computed), Wrist joint

## Abstract

**Background:**

This study aimed to evaluate the imaging performance and diagnostic value of a photon-counting detector (PCD) computed tomography (CT) compared to an energy-integrating detector (EID) and flat panel detector (FPD) for cadaveric wrist arthrographies.

**Methods:**

Following ethics committee approval, ten cadaveric wrists were injected with diluted iodinated contrast agent. CT arthrographies using PCD-, EID-, and FPD-CT were performed. Six dose protocols between 0.1 mGy (using a tin filter) and 6 mGy, ultrahigh-resolution-mode, and two reconstruction kernels were used for the PCD-CT and EID-CT. FPD-CT images were reconstructed using a “normal” and “sharp” kernel. Signal-to-noise ratios (SNR) and contrast-to-noise ratios (CNR) were calculated and analyzed using analysis of variance (ANOVA) and *post hoc* tests. Three blinded radiologists independently rated image quality concerning trabecular, cartilage, and intrinsic structures. Intraclass correlation coefficients (ICC) were calculated, followed by a Friedman and *post hoc* test.

**Results:**

At 1.5 mGy, 3 mGy, and 6 mGy with the Br89 kernel, the PCD-CT yielded up to 2.35 times higher SNR and up to 7 times higher CNR than dose-equivalent and higher dose EID-CT scans. Subjective ratings favored the PCD-CT over the EID-CT and occasionally the FPD-CT, with a combined ICC of 0.942. Applying sharper kernels, SNR did not differ significantly between the PCD-CT (1.5 mGy, 3 mGy, and 6 mGy) and the FPD-CT.

**Conclusion:**

Using sharp kernels, the PCD-CT provided superior image quality to the EID-CT and achieved comparable or better quality than the FPD at certain parameters. Thus, the PCD-CT could be considered a possible alternative in clinical routine for evaluating wrist injuries.

**Relevance statement:**

This study demonstrates the potential of the PCD-CT as a valuable tool in diagnosing wrist injuries. Its superior image quality compared to the EID-CT can increase confidence in diagnosing subtle bone pathologies and additionally yields the possibility of radiation exposure reduction.

**Key Points:**

- The technical advantages of the PCD-CT allow for dose reduction while generating high-quality images.
- PCD-CT showed superior image quality over EID-CT and was comparable to the FPD-CT.
- PDC-CT offers improved visualization of fine joint structures in wrist arthrography and should be considered in clinical routine.

**Graphical Abstract:**

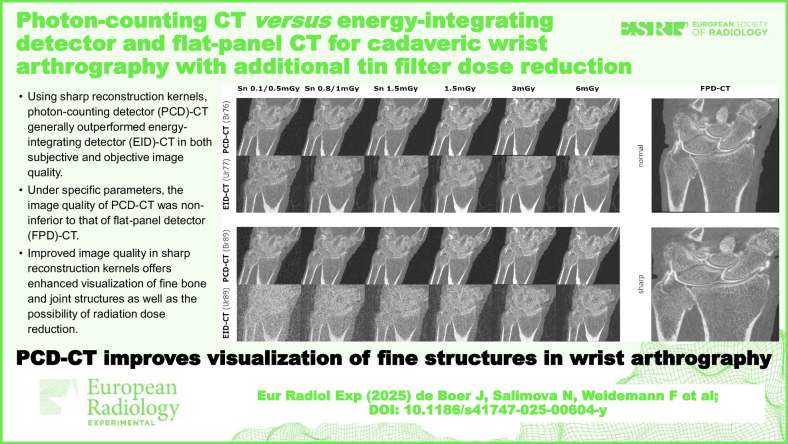

## Background

Traumatic and degenerative lesions of the wrist can lead to arthritis and chronic pain, which can significantly affect a patient´s quality of life. Due to the complex anatomy of the wrist, comprehensive and detailed imaging can be a diagnostic challenge for radiologists. Therefore, imaging techniques take a leading role in the visualization of wrist anatomy, including ligaments, trabecular bone, and cartilage. Computed tomography (CT) and magnetic resonance imaging (MRI) arthrography are well-established methods for the diagnosis and evaluation of wrist pathologies. Arthrography with multidetector CT and flat panel detector computed tomography (FPD-CT) are widely available and used methods with acquisition of high-resolution images for accurate diagnosis of small anatomical structures and compartments of the wrist. Multidetector CT arthrography is one of the most effective methods in the diagnosis of traumatic lesions of the intraosseous ligaments, cartilage structures, and bones [1]. FPD-CT arthrography is a proven method with low radiation dose when assessing musculoskeletal injuries [2–4]. Furthermore, the possibility of easy dynamic imaging is an important feature of CT arthrography when evaluating joint stability. However, CT arthrography has notable limitations in terms of assessing certain pathologies like bone marrow edema, osteonecrosis, inflammatory changes like synovitis, and internal cartilage structure. The diagnosis of ligament injuries is often limited to complete ruptures. Assessment of the ligament substance as such is often insufficient [5].

With the introduction of photon-counting detector computed tomography (PCD-CT) into clinical use, new options are available in diagnostic radiology. PCD-CT is the latest CT technology. In conventional energy-integrating detector computed tomography (EID-CT) systems, the photon energy is converted from x-rays into a light signal by a scintillator. The emitted light signals are converted into electrical signals by a photodiode. In PCD-CT, with detectors made of a semiconductor crystal, the photon energy is directly converted into electrical signals. Due to the direct energy conversion and smaller pixel sizes without optical separation, PCD-CT generates images with higher spatial resolution, reduced noise, and lower radiation dose. The higher spatial resolution is an advantage in the identification of smaller anatomical structures [6–9]. Although it does not negate the advantages of tissue contrast in MRI arthrography, a higher spatial resolution may enhance diagnostic confidence in smaller joints by improving visualization of cartilage surface irregularities and indirect signs of tissue injury, such as subtle avulsions [5].

In comparison to other CT systems, the high spatial resolution of the PCD-CT enables scanning with a lower dose for all body regions, especially in thoracic and musculoskeletal imaging [10]. Significantly improved image quality with PCD-CT in cardiovascular imaging was shown in a clinical study [11, 12]. An improved visualization of fine anatomical structures in cadaveric wrist imaging with significantly reduced radiation dose compared to dual-source CT has been reported [13, 14]. Similarly, a high-resolution and quantitative technique for the evaluation of cartilage damage in a large animal model, using PCD-CT arthrography, has been demonstrated [15]. Still, MRI remains advantageous in cartilage evaluation, as there are recent developments to establish prediction parameters based on (semi)quantitative MRI findings regarding the progression of osteoarthritis [16, 17]. Narrowing the diagnostic gap between CT and MRI, however, is especially important when an MRI is not available or contraindicated. Another important aspect in this regard is the capability of PCD-CT to provide material decomposition with virtual non-calcium imaging, which can aid in the detection of bone marrow edema [9].

Thus, the aim of this study is to evaluate the objective and subjective image quality of PCD-CT compared to EID-CT and FPD-CT in cadaveric wrist arthrography.

## Methods

This study was carried out following the ethical standards of the 1964 Declaration of Helsinki and its later amendments. The institutional review board approved the study protocol (see “Declarations”). Body donors consented to the use of their cadavers for study and research purposes during their lifetime.

### Cadaveric specimens and contrast agent application

Ten cadaveric whole arm specimens from five body donors (unknown sexes, ages, or prior wrist pathologies) were obtained from the anatomical institute of our university. Based on studies with a similar design [8, 18], as well as the use of repeated measures that reduce inter-subject variability and increase statistical power [19], the sample size was considered sufficient to demonstrate differences between the tested CT systems. A tricompartmental injection into the distal radioulnar joint, the midcarpal joint, and the radiocarpal joint was performed under fluoroscopic guidance using a 25-G needle. The contrast agent consisted of a 2:3 mixture of an iodinated agent (Iomeprol 300 mg/mL, Iomeron 300, Bracco Imaging) and NaCl. The 5–8-mL contrast mixture was injected into each wrist.

### FPD-CT arthrography

Following the intra-articular injection, an FPD-CT arthrography was acquired using the same angiographic system (ARTIS pheno, Siemens Healthineers AG). Prior to the scans, a dose measurement was performed to verify the comparability of the protocols. By means of a three-dimensional printer (Formlabs GmbH), a human wrist-sized phantom was printed with a central aperture for placement of a dose measuring chamber. For predefined settings of the FPD-CT (70 kVp, 555.52 mAs, no additional filter) and the PCD-CT (120 kVp, 100 mAs, 10-mm Al filter), radiation doses of 19.97 mGy and 25.30 mGy were measured by L.B. and T.W., suggesting an approximate comparability of the CT systems. Thus, for the FPD-CT, a scan protocol used in clinical routine was applied. Tube voltage was 70 kVp, and tube current was 315 mA. The average dose-area product was 343.76 µGym². Images were reconstructed using the smallest possible volume-of-interest per wrist and “normal”/“sharp” bone reconstruction kernel. The resulting voxel size was in the range of 0.11–0.12 mm.

### PCD- and EID-CT arthrography

After transfer to the CT units, images were acquired using a clinical PCD-CT system (NAEOTOM Alpha, Siemens Healthineers AG) and a dual-source EID-CT scanner (SOMATOM Force, Siemens Healthineers AG). Initially, three different dose protocols were selected with a fixed reference tube voltage of 120 kVp in single-energy mode. Tube currents were adjusted according to the volume CT dose index−CTDI_vol_ to result in a low-dose (1.5 mGy), standard-dose (3 mGy), and high-dose (6 mGy) scan protocol. For an additional low-dose (1.5 mGy) scan, a tin filter was applied to both scanners. Lastly, two more ultralow-dose scans with the additional tin filter were performed: 0.8 mGy and 0.1 mGy for the PCD-CT and 1.0 mGy and 0.5 mGy for the EID-CT. For the EID-CT, a further dose reduction did not produce reasonable images; hence, 0.5 mGy was chosen as the lowest value. This range of doses was chosen to reflect a broad spectrum of different clinical scenarios, including pediatric imaging or precise preoperative planning. The additional ultralow-dose protocols and the tin filter were implemented to test lower limits of radiation dose and (visually) emphasize the theoretical potential for dose reduction in the PCD-CT. A tin filter hardens the x-ray spectrum by filtering out low-energy photons, as these photons rather contribute to the dose than to image quality. Hardening the beam, however, increases noise and reduces soft tissue contrast, which might decrease diagnostic accuracy when assessing subtle joint injuries [20, 21]. For all scans, the pitch factor was 0.8, and the rotation time was 1,000 ms.

### Reconstruction and post-processing

**Fig. 1:**
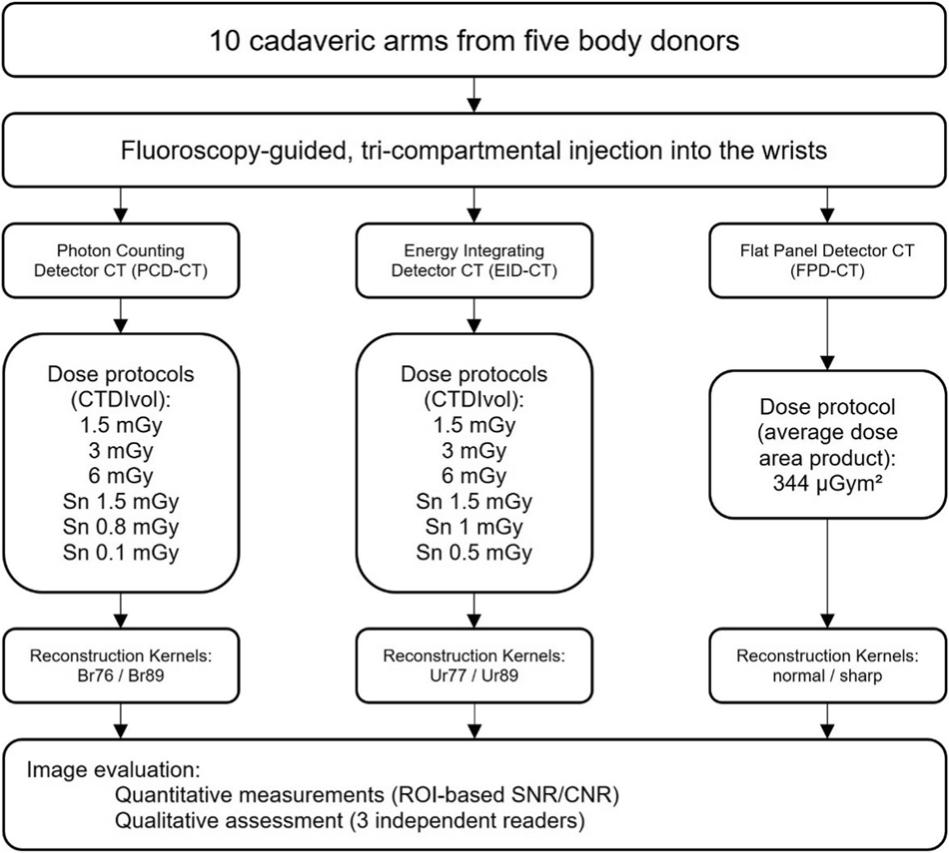
Flowchart of the study setup. CNR, Contrast-to-noise ratio; CT, Computed tomography; CTDI_vol_, Volume CT dose index; EID-CT, Energy-integrating detector CT; FPD-CT, Flat-panel detector CT; PCD-CT, Photon-counting detector CT; ROI, Region-of-interest; SNR, Signal-to-noise ratio

The imaging data of the PCD-CT and EID-CT were reformatted with a field of view of 100 mm and an image matrix of 512 × 512 to ensure an identical in-plane resolution. Slice-thickness and increment were chosen as small as technically possible for each scanning mode. Different reconstruction kernels, including sharp kernels, were compared for each scanner. The flowchart shows the study setup (Fig. 1). Scan parameters for all CT systems are summarized in Table 1.

**Table 1:** Scan protocols and reconstruction parameters of all three CT systems

	PCD-CT		EID-CT		Flat-panel detector CT
Scanner	NAEOTOM Alpha		SOMATOM Force		ARTIS Pheno
	No filter	Tin filter	No filter	Tin filter	No filter
CTDI_vol_ (mGy)	1.5 mGy (19 mAS)	1.5 mGy (176 mAs)	1.5 mGy (26 mAs)	1.5 mGy (400 mAs)	DAP: 344 µGym² (average)
	3 mGy (38 mAs)	0.8 mGy (92 mAs)	3 mGy (52 mAs)	1 mGy (265 mAs)	–
	6 mGy (75 mAs)	0.1 mGy (13 mAs)	6 mGy (103 mAs)	0.5 mGy (132 mAs)	–
Collimation	24/0.2	24/0.2	19.2/0.6	19.2/0.6	Rectangular (1/976/1/976)
Rotation time (s/rotation)	1	1	1	1	6
Pitch factor	0.8	0.8	0.8	0.8	–
Slice thickness (mm)	0.2	0.2	0.4	0.4	0.2
Increment (mm)	0.1	0.1	0.2	0.3	0.2
Field of view (mm)	100 × 100	100 × 100	100 × 100	100 × 100	100 × 100
Matrix size	512 × 512	512 × 512	512 × 512	512 × 512	512 × 512
Reconstruction	Br89	Br89	Ur89	Ur89	Normal
Kernel	Br76	Br76	Ur77	Ur77	Sharp

*CT* Computed tomography, *CTDIvol* Volume CT dose index, *DAP* Dose-area product

### Subjective image quality assessment

Two independent radiologists with more than seven years in practice and one radiologist with less than one year in practice evaluated all CT images. The radiologists were blinded to identifying information on the images. Images were evaluated on a client-server-based picture archiving and communication system workstation (Visage 7.1, Visage Imaging GmbH, Berlin, Germany). The readers were allowed to change the initial window settings of 100/3,500 HU (contrast/width) to their requirements. The reader´s instructions were to evaluate the visibility of intrinsic articular structures (*e.g*., ligaments, triangular fibrocartilage complex), trabecular structures and cartilage and rate the image quality on a 7-point scale as follows: 1 = insufficient image quality (nondiagnostic), 2 = very poor, 3 = poor, 4 = satisfactory, 5 = good, 6 = very good, 7 = excellent image quality.

### Objective image quality assessment

**Fig. 2:**
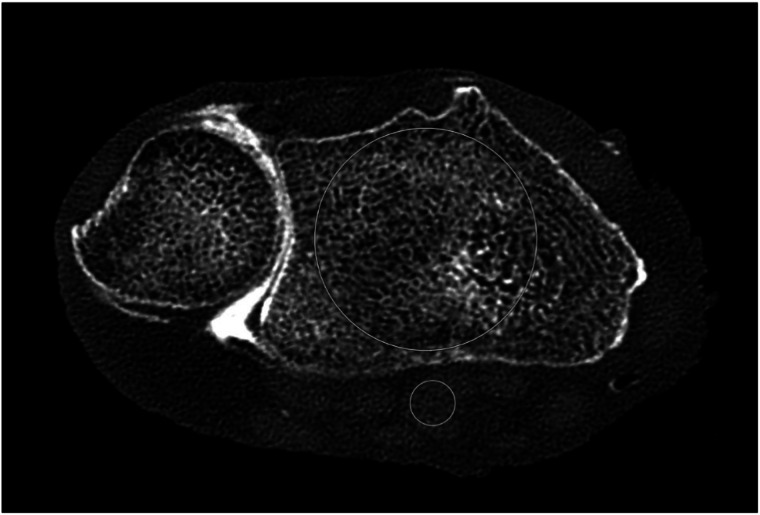
Exemplary image of the ROI placement within the distal radius (big circle) and an adjacent muscle (small circle)

Within three adjacent CT slices, two regions of interest were placed. One was placed inside the radial trabecular bone, the other one within a surrounding muscle (Fig. 2). For each region-of-interest (ROI), the mean signal attenuation and standard deviation were extracted in HU. Signal-to-noise ratio (SNR) and contrast-to-noise ratio (CNR) were calculated using the following formulas:

$${{\rm{SNR}}}={{{\rm{HU}}}}_{{{\rm{bone}}}}/{{{\rm{SD}}}}_{{{\rm{bone}}}}$$

$${{\rm{CNR}}}=({{{\rm{HU}}}}_{{{\rm{bone}}}}-{{{\rm{HU}}}}_{{{\rm{muscle}}}})/{{{\rm{SD}}}}_{{{\rm{bone}}}}$$

### Statistical analysis

All statistical analyses were calculated with SPSS Statistics 27.0.0.0 (IBM Corp., Armonk, New York, USA). The “sharp” kernel of the FPD-CT was compared to the Br89/Ur89 kernel of the PCD-CT and EID-CT. Accordingly, the “normal” kernel of the FPD-CT was compared to the Br76/Ur77 of the PCD-CT and EID-CT. Furthermore, SNR and CNR values of the PCD-CT´s Br89 were compared to those of the EID-CT´s Ur77 and the FPD-CT´s normal kernel.

The null hypothesis stated that there is no difference in subjective and objective image quality between the three scanners. Reliability between raters was calculated using the intraclass correlation coefficient (ICC) for all measurements in the two-way, mixed, consistency model [22]. The ICC values were interpreted as follows: values ≤ 0.4 were interpreted as bad, 0.4–0.59 as intermediate, 0.60–0.74 as good, and 0.75–1.00 as very good correlation [23]. For comparison of the subjective ratings for different scan variables, Friedman´s one-way analysis of variance (ANOVA) by rank, followed by a post hoc test to correct for multiple comparisons, was performed since the Kolmogorov–Smirnov test showed no normal distribution. We considered *p*-values lower than 0.05 statistically significant. Concerning the SNR and CNR measurements, the Kolmogorov–Smirnov test showed a normal distribution of the FPD-CT, EID-CT, and PCD-CT measurements. Thus, a one-way ANOVA with repeated measures followed by the Tukey *post hoc* test was performed. We considered *p*-values lower than 0.05 statistically significant.

## Results

**Fig. 3:**
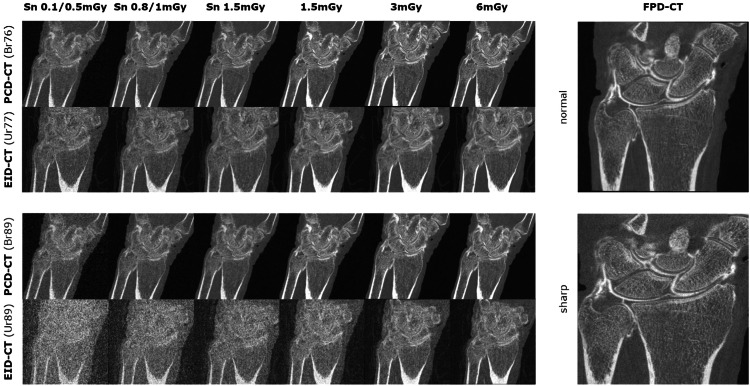
Exemplary images of all dose parameters and reconstruction kernels in the PCD-CT, EID-CT, and FPD-CT. CT, Computed tomography; EID-CT, Energy-integrating detector CT; FPD-CT, Flat-panel detector CT; PCD-CT, Photon-counting detector CT

Ten wrists were each scanned with six different protocols using the PCD-CT and the EID-CT. One protocol with two different kernel reconstructions was used for the FPD-CT. This resulted in 130 individual examinations. Due to technical issues with the PCD-CT, only nine out of the ten cadaveric wrists could be included in its analysis. Representative images are presented in Fig. 3.

### Objective image quality assessment

For all dose protocols, there was no significant statistical difference in SNR or CNR for the Br76/Ur77 comparison between the PCD-CT and EID-CT. However, the PCD-CT´s SNR and CNR were significantly higher in the Br89/Ur89 comparison at 1.5 mGy, 3 mGy, and 6 mGy. Additionally, the PCD-CT´s CNR was significantly higher in the Br89/Ur89 comparison at 0.8/1 mGy. The remaining ultralow-dose comparisons showed no statistically significant difference. When comparing the PCD-CT´s Br89 kernel to the EID-CT´s Ur77 at dose equivalents, there was no statistically significant difference in SNR and CNR (*p* = 0.646–0.998). Throughout all dose protocols, SNR and CNR were significantly higher for the FPD-CT´s “normal” kernel than for the PCD-CT´s Br89, with *p*-values ranging from < 0.001 to 0.002.

Comparing the PCD-CT to the FPD-CT, there was no significant difference in SNR for the Br89/”sharp” comparison at 1.5 mGy, 3 mGy, and 6 mGy. Additionally, there was no difference in CNR at 6 mGy/Br89/”sharp”. For all other parameters, the FPD-CT showed significantly higher SNR and CNR values compared to the PCD-CT.

**Fig. 4:**
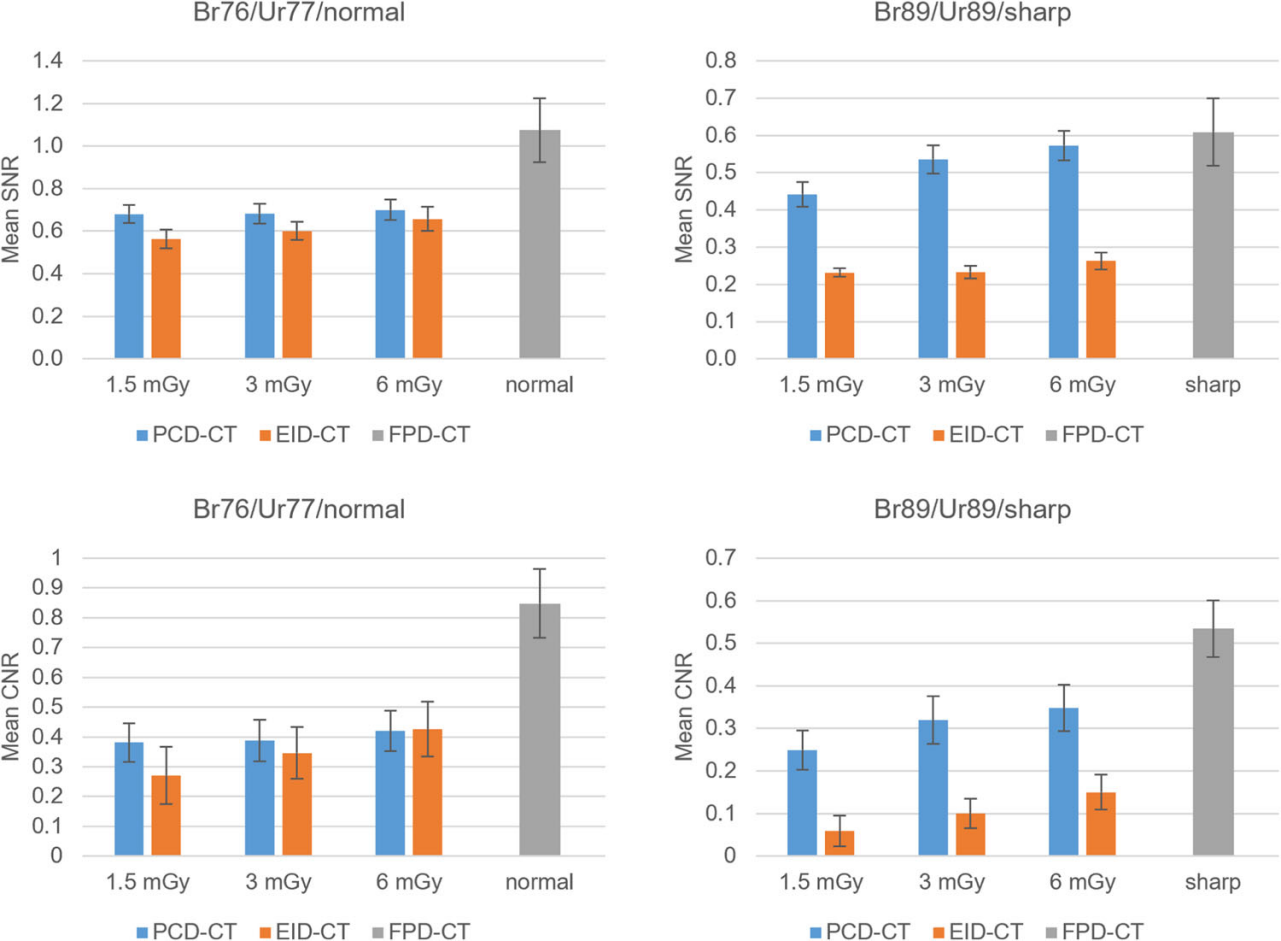
Mean SNR and CNR for different dose protocols and reconstruction kernels. Br76 of the PCD-CT was compared to Ur77 of the EID-CT and “normal” of the FPD-CT. Br89 of the PCD-CT was compared to Ur89 of the EID-CT and “sharp” of the FPD-CT. CNR, Contrast-to-noise ratio; CT, Computed tomography; EID-CT, Energy-integrating detector CT; FPD-CT, Flat-panel detector CT; PCD-CT, Photon-counting detector CT; SNR, Signal-to-noise ratio

**Fig. 5:**
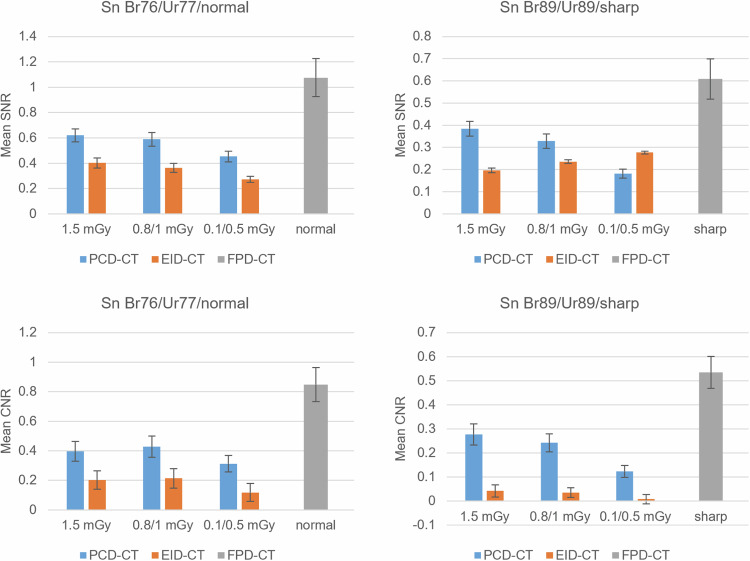
Mean SNR and CNR for the different low-dose protocols and reconstruction kernels. A tin filter was used for both the PCD-CT and the EID-CT. Br76 of the PCD-CT was compared to Ur77 of the EID-CT and “normal” of the FPD-CT. Br89 of the PCD-CT was compared to Ur89 of the EID-CT and “sharp” of the FPD-CT. CNR, Contrast-to-noise ratio; CT, Computed tomography; EID-CT, Energy-integrating detector CT; FPD-CT, Flat-panel detector CT; PCD-CT, Photon-counting detector CT; SNR, Signal-to-noise ratio

**Fig. 6:**
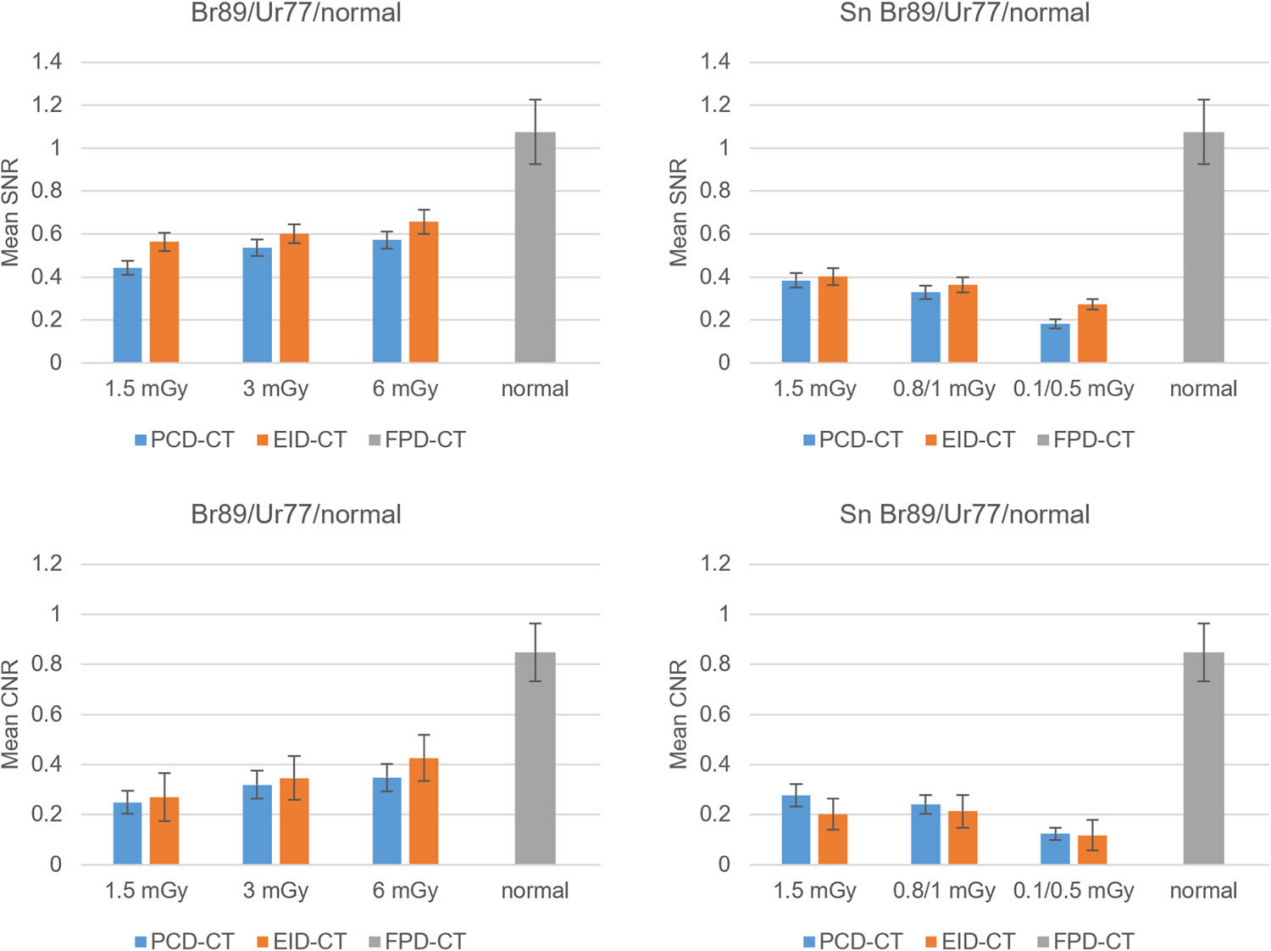
Mean SNR and CNR for the different dose protocols and reconstruction kernels. A tin filter was used for both the PCD-CT and the EID-CT low-dose protocols. Br89 of the PCD-CT was compared to Ur77 of the EID-CT and “normal” of the FPD-CT. CNR, Contrast-to-noise ratio; CT, Computed tomography; EID-CT, Energy-integrating detector CT; FPD-CT, Flat-panel detector CT; PCD-CT, Photon-counting detector CT; SNR, Signal-to-noise ratio

Comparing the EID-CT with the FPD-CT, the SNR and CNR of the FPD-CT were significantly higher for all measured comparisons. Detailed results are presented in Supplemental Table S1 and Figs. 4–6.

### Subjective image quality assessment

ICC for all CT systems combined measured 0.942, showing a very good interrater correlation. ICC was 0.917 for the PCD-CT and 0.912 for the EID-CT. With an ICC of only 0.382, the FPD-CT showed poor interreader agreement. In total, 308 of the 972 PCD-CT assessments (31.7%) were rated good, very good, or excellent; only 136 (14%) were rated nondiagnostic, and 44 (4.5%) were rated as excellent.

In total, 870 of the 1,080 EID-CT assessments (80.6%) were rated as insufficient or very poor; only 73 (6.8%) were rated satisfactory. The highest achieved rating for the EID-CT was good image quality, which was only awarded 15 of 1,080 times (1.4%). From 0.5 mGy to 1.5 mGy, 100% of the EID-CT ratings were nondiagnostic when using the sharp kernel. With the high-dose protocol (6 mGy) and a sharp kernel, 59 of 90 (65.6%) of ratings were nondiagnostic. Using the “smooth” kernel, 66 of 90 (73.3%) of ratings were nondiagnostic at 0.5 mGy, while none were nondiagnostic at 6 mGy.

**Fig. 7:**
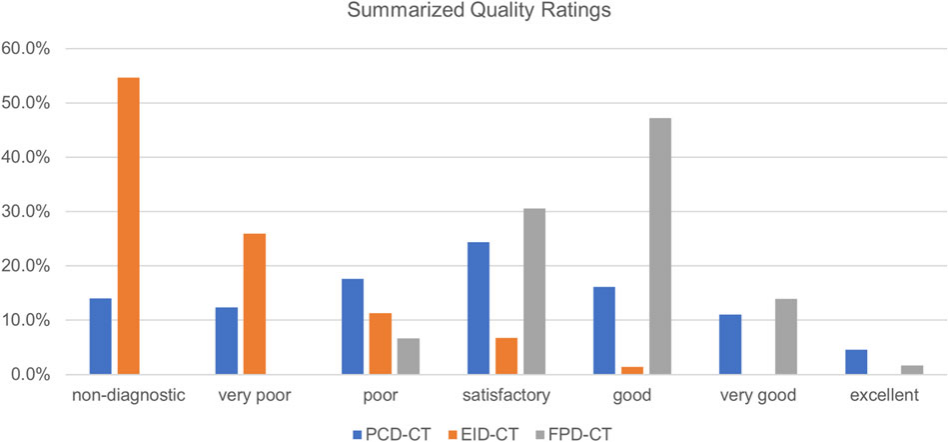
Cumulated relative values of the 7-point rating scale quality assessment. CT, Computed tomography; EID-CT, Energy-integrating detector CT; FPD-CT, Flat-panel detector CT; PCD-CT, Photon-counting detector CT

The FPD-CT achieved an excellent rating 3 of 180 times (1.7%). A good or very good rating was given in 110 of 180 cases (61.1%). Detailed grading features are represented in the Supplemental Table S2. Summarized quality ratings are represented in Table 2 and Fig. 7.

**Table 2:** Absolute and relative frequencies of ratings for the three CT systems on a 7-point rating scale

Rating value	PCD-CT	EID-CT	Flat-panel detector CT
1	136 (13.99%)	590 (54.63%)	0 (0.0%)
2	120 (12.35%)	280 (25.93%)	0 (0.0%)
3	171 (17.59%)	122 (11.3%)	12 (6.67%)
4	237 (24.38%)	73 (6.76%)	55 (30.56%)
5	157 (16.15%)	15 (1.39%)	85 (47.22%)
6	107 (11.01%)	0 (0.0%)	25 (13.89%)
7	44 (4.53%)	0 (0.0%)	3 (1.67%)

Values for all dose protocols, readers, and rated structures are combined

*CT* Computed tomography

Except for the ultralow-dose comparison (0.1 mGy and 0.5 mGy), the PCD-CT achieved significantly better ratings than the EID-CT at dose-equivalents for all protocols and kernels when evaluating intrinsic, trabecular, and cartilage structures. At 1.5 mGy, with Br89, the PCD-CT´s ratings were still significantly higher compared to the EID-CT´s Ur89 at 3 mGy or even 6 mGy (*p* < 0.001).

At 3 mGy and 6 mGy, PCD-CT consistently showed no inferiority throughout all rated structures to the FPD-CT when comparing Br76 to the “normal” kernel and Br89 to the “sharp” kernel. In fact, at 6 mGy/Br76, the PCD-CT´s visualization of intrinsic and trabecular structures, as well as the trabecular structures at 6 mGy/Br89, were rated significantly higher than the FPD-CT´s. In some cases, the PCD-CT showed no inferiority at 1.5 mGy when compared to the FPD-CT. When further lowering the doses of the PCD-CT, however, the FPD-CT produced significantly better ratings.

Comparing the EID-CT with the FPD-CT, almost all ratings were higher for the FPD-CT. The ratings, however, for the trabecular structures when comparing the EID-CT´s Ur77 at 6 mGy to the FPD-CT´s “normal” kernel were not significantly lower.

**Fig. 8:**
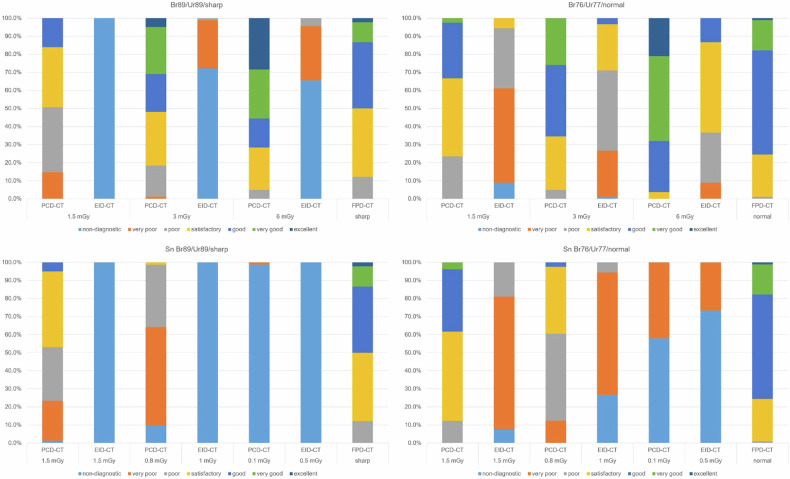
Cumulated quality assessment on a 7-point rating scale for all three readers, 10 arms, and rated structures according to different dose protocols and reconstruction kernels. CT, Computed tomography; EID-CT, Energy-integrating detector CT; FPD-CT, Flat-panel detector CT; PCD-CT, Photon-counting detector CT

Detailed results are presented in Supplemental Tables S3–S5 and Fig. 8.

## Discussion

In this study, we examined the objective and subjective image quality of wrist arthrography at different x-ray dosages using the PCD-CT and compared it to an EID-CT and an FPD-CT. Applying sharper kernels, both SNR and CNR were significantly higher for the PCD-CT when compared to the dose-equivalent and even the higher doses of the EID-CT. In addition, the subjective ratings by three independent radiologists were significantly higher for the PCD-CT than for the EID-CT in almost all compared scanning protocols.

At 1.5 mGy, 3 mGy, and 6 mGy, the sharper kernel of the PCD-CT did not show significantly lower SNR than the FPD-CT with the “sharp” kernel. Furthermore, in mid to high range doses (3, 6 mGy), subjective ratings of the PCD-CT were either non-inferior or, in some cases, even superior to the FPD-CT.

Our findings are supported by existing literature. In recent studies, PCD-CT has been shown to generate images with higher spatial resolution, reduced noise, and better SNR and CNR compared to the EID-CT [8, 13, 14, 18, 24–28]. This was even observed when reducing the radiation doses of the PCD-CT. Importantly, this is not exclusive to musculoskeletal imaging. Boccalini et al [12] found improved objective and subjective image quality when assessing coronary stents, even at reduced radiation doses. A possible clinical relevance in wrist imaging has already been discussed in recent studies [29]. The PCD-CT´s smaller pixel electrode size helps achieve images with higher spatial resolution than the EID-CT without the need for additional comb filters. The unnecessary use of comb filters, as well as interpixel septae, increases the dose efficiency of the PCD-CT and therefore offers the opportunity of dose reduction [14, 24, 28, 30–32].

Various studies have also reported a significant dose reduction while maintaining image quality using a tin filter [20, 21, 33, 34]. Although the dose reduction can cause an increase in noise, Stern et al [20] and Braun et al observed a higher dose efficiency compared to standard protocols [33]. Stern et al [20] also found that despite a 6.1-fold dose reduction, the tin-filtered low-dose CT provided an image quality sufficient to assess bone anatomy and pathologies.

With the added tin filter, there was no significant difference in SNR and CNR between the PCD-CT and the EID-CT except for the Br89/Ur89 comparison at 1.5 mGy and 0.8/1 mGy. Still, our readers preferred almost all tin-filtered images of the PCD-CT over those of the EID-CT. Considering the technical advantages of the PCD-CT and the mechanism of the tin filter, an argument for a possible complementation in dose reduction can be made.

Image sharpness is essential in imaging of small joints and when identifying minuscule anatomical structures and pathologies. When choosing reconstruction kernels, sharpness and noise must be balanced well since sharper kernels come at the expense of a decreased CNR and SNR [18, 27]. This primarily became apparent in our subjective ratings. While most EID-CT exams at Br89 were rated “nondiagnostic” or “very poor”, the PCD-CT generated mostly positive ratings. When applying sharp kernels, the PCD-CT even achieved substantially higher ratings at 1.5 mGy than the EID-CT did at 3 mGy and 6 mGy. Since the FPD-CT images were predominantly rated between satisfactory and very good, determining the threshold for sufficiency may have been more challenging for readers. Unlike the other CT systems, the FPD-CT generated no clearly nondiagnostic images, possibly resulting in greater disagreement. This may help explain the relatively low interreader agreement (ICC = 0.382).

The fact that there was no statistically significant difference in SNR and CNR when comparing the PCD-CT´s Br89 to the EID-CT´s Ur77 at dose equivalents underlines the PCD-CT´s potential for noise reduction. Usually, low-energy pulses create electronic noise. Since the PCD-CT converts the incoming x-ray beams into pulses that are only counted if they exceed a certain threshold, even at low radiation doses, noise can be effectively removed [30]. As discussed by Kämmerling et al, this opens the possibility of using sharper kernels despite usually higher noise levels [27].

Additionally, the PCD-CT weighs high- and low-energy photons equally, as opposed to the EID-CT, which allows for better contrast, with or without contrast agent [30].

As an addition to most existing studies, we performed a CT arthrography, which allowed us to assess cartilage structures, create an overall better contrast, and extend the comparison to an FPD-CT. Kirschke et al [35] demonstrated the clinical value of CT arthrography for evaluation of osteochondral lesions at the Ankle. They reported a substantially higher detection rate of full-thickness and intraoperatively confirmed cartilage lesions of the ankle joint compared to the MRI. Similarly, various other studies have reported a superiority of the CT arthrography for detection of osteochondral lesions of the knee or ankle compared to conventional MRI and even MRI arthrography [36, 37]. Cha et al also concluded that MRI alone may not be sufficient to detect osteochondral lesions of the talus, due to its low sensitivity (46%) and low interobserver reliability [38]. Even more so, this might apply to the wrist being composed of more and smaller bones and therefore having more surface area for osteochondral lesions. This underlines the clinical importance of CT arthrography, since it has the potential to directly impact therapeutic decisions and offers a viable option for perioperative diagnostics, especially for cases with metal implants [35]. A potential application in wrist imaging is the postoperative assessment of scaphoid nonunion following screw osteosynthesis. However, given the CT´s inherent limitations in soft tissue and internal cartilage evaluation, the PCD-CT, despite its advantages over the EID-CT, remains complementary to the MRI and MR arthrography. Although both modalities are usually performed together, advancements in CT image quality are still important as they might make a difference in therapeutic decisions. The advantages of the PCD-CT, such as higher spatial resolution, might become more apparent in smaller joints or cases of subtle bony injuries. Better visualization of cartilage surface irregularities or indirect signs of ligament injuries, such as small avulsions, also becomes especially important when MRI is not available or contraindicated.

When performing arthrography, a crucial advantage of the FPD-CT is the convenience of the procedure. Being able to perform a CT scan instantly after fluoroscopically assisted injection without the need for patient transfer drastically improves the workflow [37, 39]. However, it is important to note that the FPD-CT is primarily reserved for smaller joints. Benefits such as high spatial resolution or a good SNR are particularly evident in small scan volumes. When scanning bigger, more proximal joints such as the shoulder or hip, the contralateral joint, as well as the incomplete trajectory of the C-arm, lead to image impairment and truncation artifacts in the peripheral areas of the field of view. A recent study reported a low (40%) sensitivity for detecting cartilage pathologies in low-dose FPD-CT arthrographies of the shoulder and a moderate (75%) sensitivity, whilst using a considerably higher radiation dose compared to a multidetector CT arthrography [40]. This demonstrates the FPD-CT´s noise dependence on radiation dose. Further decrease of sensitivity is caused by its susceptibility to various artifacts, such as cone beam artifacts [40]. Therefore, the application of FPD-CT arthrography should be critically evaluated, especially in patients with frequent follow-ups. Since scenarios with frequent follow-ups also often involve metal implants, the PCD-CT´s ability to reduce metal-artifact reduction, as discussed by Chang et al [41], might prove beneficial. The potential for arthrographies was also shown by Rajendran et al [15]: utilizing its high resolution and multienergy imaging capabilities, these authors demonstrated an arthrography technique to reliably assess joint disease. In general, a high resolution and a sufficient contrast between the contrast agent and the surrounding tissue are vital for detecting small cartilage lesions. The aforementioned features of the PCD-CT yield the possibility of notable dose reduction compared to the EID-CT and FPD-CT, making it a promising modality for multiple clinical scenarios such as young patients, frequent follow-ups, or unavailable or contraindicated MRI.

Limitations of this study should be considered as follows. Firstly, only ten cadaveric wrists were assessed without information concerning the age, condition of the bone, or previous injuries. This limits the generalizability of our results to a broader patient population. Furthermore, while efforts were made to achieve a homogenous contrast in all ten wrists, the different degrees of tissue- and joint capsule alterations of the cadaveric specimen, as well as the movement of the wrists and latency during transfer between scanners may have influenced the distribution of the contrast media and therefore the overall image. The comparability of different iterative reconstruction algorithms is an additional limitation to this study.

The reconstruction kernels were selected to be as comparable as possible in terms of image sharpness, but they were not identical, especially since the FPD-CT contributed only two different selectable reconstruction kernels. CT scanners of only one manufacturer were investigated for accessibility reasons. Furthermore, the limited comparability of dose protocols of the FPD-CT must be mentioned, particularly as the used scanner model is not capable of providing a CTDI value. The experimental measurement by means of a wrist phantom suggested closest comparability of the applied FPD-CT scanner settings to the high-dose protocols (6 mGy) of the PCD-CT (75 mAs) and EID-CT (103 mAs) scanners.

In conclusion, our results contribute to the growing evidence that the PCD-CT provides the possibility of overall dose reduction without quality loss, given its superior image quality compared to the EID-CT and reduced noise in sharper kernels. While simultaneously providing on-par image quality with the FPD-CT at certain parameters, the PCD-CT ought to be discussed as a viable alternative when performing wrist arthrography.

## Supplementary information

**Additional file 1:** **Table S1.** SNR and CNR calculations. **Table S2.** Detailed grading features. **Table S3.** Detailed quality assessment and analysis of intrinsic structures. **Table S4.** Detailed quality assessment and analysis of trabecular structures. **Table S5.** Detailed quality assessment and analysis of cartilage structures.

## Data Availability

All used datasets are available from the corresponding author on request.

## Abbreviations

CNR: Contrast-to-noise ratio
CT: Computed tomography
EID-CT: Energy-integrating detector CT
FPD-CT: Flat-panel detector CT
ICC: Intraclass correlation coefficient
MRI: Magnetic resonance imaging
PCD-CT: Photon-counting detector CT
ROI: Region-of-interest
SNR: Signal-to-noise ratio
