# Supplementary material for: Photon-counting CT versus energy-integrating detector and flat-panel CT for cadaveric wrist arthrography with additional tin filter dose reduction
Source: Eur Radiol Exp. 2025 Aug 29;9:83. doi: 10.1186/s41747-025-00604-y (PMC12397008; doi:10.1186/s41747-025-00604-y)
Supplement: Supplementary file 1 — Additional file 1: Table S1. SNR and CNR calculations. Table S2. Detailed grading features. Table S3. Detailed quality assessment and analysis of intrinsic structures. Table S4. Detailed quality assessment and analysis of trabecular structures. Table S5. Detailed quality assessment and analysis of cartilage structures. [file 41747_2025_604_MOESM1_ESM.pdf]

Photon-counting CT versus energy-integrating detector and flat-panel CT for cadaveric wrist arthrography with additional tin filter dose reduction

ELECTRONIC SUPPLEMENTARY MATERIAL

Table S1. SNR and CNR calculations

	Kernel	Filter	Dose protocol	Scanner: PCD-CT (mean ± SEM)	Scanner: EID-CT (mean ± SEM)	Scanner: FPD-CT[1] (mean ± SEM)	*p*-values: Combined	*p*-values: PCD vs. EID	*p*-values: PCD vs. FPD	*p*-values: EID vs. FPD
SNR	Br76/Ur77/normal	/	1.5 mGy	0.68 ± 0.04	0.56 ± 0.04	1.08 ± 0.15	0.001	0.672	0.014	0.001
			3 mGy	0.68 ± 0.05	0.6 ± 0.04	1.08 ± 0.15	0.001	0.825	0.015	0.002
			6 mGy	0.7 ± 0.05	0.66 ± 0.06	1.08 ± 0.15	0.006	0.95	0.026	0.009
		Sn	1.5 mGy	0.62 ± 0.05	0.4 ± 0.04	1.08 ± 0.15	<0.001	0.261	0.004	<0.001
			0.8/1 mGy	0.59 ± 0.05	0.36 ± 0.03	1.08 ± 0.15	<0.001	0.233	0.002	<0.001
			0.1/0.5 mGy	0.45 ± 0.04	0.27 ± 0.02	1.08 ± 0.15	<0.001	0.366	<0.001	<0.001
	Br89/Ur89/sharp	/	1.5 mGy	0.44 ± 0.03	0.23 ± 0.01	0.61 ± 0.09	<0.001	**0.032**	**0.111**	<0.001
			3 mGy	0.54 ± 0.04	0.23 ± 0.02	0.61 ± 0.09	<0.001	**0.02**	**0.658**	<0.001
			6 mGy	0.57 ± 0.04	0.26 ± 0.02	0.61 ± 0.09	<0.001	**0.001**	**0.907**	<0.001
		Sn	1.5 mGy	0.38 ± 0.03	0.2 ± 0.01	0.61 ± 0.09	<0.001	0.064	0.021	<0.001
			0.8/1 mGy	0.33 ± 0.03	0.24 ± 0.01	0.61 ± 0.09	<0.001	0.493	<0.001	<0.001
			0.1/0.5 mGy	0.18 ± 0.02	0.28 ± 0.01	0.61 ± 0.09	<0.001	0.462	<0.001	<0.001

CNR	Br76/Ur77/normal	/	1.5 mGy	0.38 ± 0.06	0.27 ± 0.1	0.85 ± 0.12	<0.001	0.679	0.003	<0.001
			3 mGy	0.39 ± 0.07	0.35 ± 0.09	0.85 ± 0.12	<0.001	0.949	0.003	0.001
			6 mGy	0.42 ± 0.07	0.43 ± 0.09	0.85 ± 0.12	0.002	0.999	0.006	0.006
		Sn	1.5 mGy	0.4 ± 0.07	0.2 ± 0.06	0.85 ± 0.12	<0.001	0.261	0.001	<0.001
			0.8/1 mGy	0.43 ± 0.07	0.21 ± 0.07	0.85 ± 0.12	<0.001	0.211	0.004	<0.001
			0.1/0.5 mGy	0.31 ± 0.06	0.12 ± 0.06	0.85 ± 0.12	<0.001	0.241	<0.001	<0.001
	Br89/Ur89/sharp	/	1.5 mGy	0.25 ± 0.05	0.06 ± 0.04	0.53 ± 0.07	<0.001	**0.03**	0.001	<0.001
			3 mGy	0.32 ± 0.06	0.1 ± 0.03	0.53 ± 0.07	<0.001	**0.014**	0.017	<0.001
			6 mGy	0.35 ± 0.05	0.15 ± 0.04	0.53 ± 0.07	<0.001	**0.036**	**0.051**	<0.001
		Sn	1.5 mGy	0.28 ± 0.04	0.04 ± 0.03	0.53 ± 0.07	<0.001	**0.03**	0.001	<0.001
			0.8/1 mGy	0.24 ± 0.04	0.04 ± 0.02	0.53 ± 0.07	<0.001	**0.006**	<0.001	<0.001
			0.1/0.5 mGy	0.12 ± 0.02	0.01 ± 0.02	0.53 ± 0.07	<0.001	0.149	<0.001	<0.001

SNR and CNR Calculations for all dose protocols, filter used and reconstruction kernels using the ROI-based measurement of signal attenuation. Mean values and the standard error of mean (±SEM) are given. The *p*-values of the Tukey post hoc analysis as well as the combined *p*-value of the ANOVA is reported. *p*-values for significant differences between PCD-CT and EID-CT as well as non-significant differences between PCD-CT and FPD-CT are highlighted in bold. *ANOVA* Analysis of variance, *CNR* Contrast-to-noise ratio, *CT* Computed tomography, *EID-CT* Energy-integrating detector CT, *FPD-CT* Flat-panel detector CT, *PCD-CT* Photon-counting detector CT, *ROI* Region-of-interest, *SNR* Signal-to-noise ratio, *SEM* Standard error of mean.

[1] Added data for a better overview. The FPD-CT did not provide different dose protocols or a tin filter

Table S2 Detailed grading features

**Rating**	**Intrinsic structures***	**Trabecular structures**	**Cartilage structures**
Nondiagnostic	Very high noise, no visible distinction between anatomical structures	Very high noise, no visible distinction between anatomical structures	Very high noise, no visible distinction between anatomical structures
Very poor	Blurred structures	Trabeculae not clearly discernible	Blurred surfaces, cartilage not identifiable
Poor	Structures barely visible	Trabeculae barely identifiable	Cartilage surface barely discernible
Satisfactory	Clear anatomical structures, low contrast	Trabecular structures visible, low contrast	Adequate delineation of cartilage surface
Good	Well defined structures, some noise	Good visualization of trabecular structures, good contrast	Smooth cartilage surfaces, good contrast
Very good	Sharp delineation, minimal noise	Fine trabecular structures visible	Sharply defined cartilage, smaller irregularities identifiable
Excellent	Excellent delineation, almost no noise	Very fine trabecular details, excellent contrast	Excellent delineation of the cartilage

Detailed grading features for the three independent readers to assess image quality

*Such as scapholunate ligament, triangular fibrocartilage complex, lunotriquetral ligament.

Table S3 Detailed quality assessment and analysis of intrinsic structures

			**Intrinsic structures rating value (mean [min−max])**			***p*-values**			
Kernel	Filter	Dose protocol	PCD-CT	EID-CT	FPD-CT[1]	Combined	PCD vs. EID	PCD vs. FPD	EID vs. FPD
Br76/Ur77/normal	/	1.5 mGy	3.67 (3-4)	2.33 (1-4)	5 (4-7)	<0.001	**0.001**	0.003	<0.001
		3 mGy	4.59 (3-6)	2.9 (2-4)	5 (4-7)	<0.001	**<0.001**	**0.922**	<0.001
		6 mGy	5.81 (4-7)	3.47 (2-5)	5 (4-7)	<0.001	**<0.001**	**0.035**	<0.001
	Sn	1.5 mGy	4.3 (3-6)	2.03 (1-3)	5 (4-7)	<0.001	**<0.001**	**0.231**	<0.001
		0.8/1 mGy	3.33 (2-5)	1.77 (1-3)	5 (4-7)	<0.001	**0.001**	0.002	<0.001
		0.1/0.5 mGy	1.44 (1-2)	1.23 (1-2)	5 (4-7)	<0.001	1	<0.001	<0.001
Br89/Ur89/sharp	/	1.5 mGy	3.48 (2-5)	1 (1-1)	4.57 (3-7)	<0.001	**<0.001**	**0.088**	<0.001
		3 mGy	4.41 (3-7)	1.27 (1-2)	4.57 (3-7)	<0.001	**<0.001**	**1**	<0.001
		6 mGy	5.37 (3-7)	1.37 (1-3)	4.57 (3-7)	<0.001	**<0.001**	**0.231**	<0.001
	Sn	1.5 mGy	3.26 (2-5)	1 (1-1)	4.57 (3-7)	<0.001	**<0.001**	0.019	<0.001
		0.8/1 mGy	2.3 (1-4)	1 (1-1)	4.57 (3-7)	<0.001	**0.003**	0.001	<0.001
		0.1/0.5 mGy	1.04 (1-2)	1 (1-1)	4.57 (3-7)	<0.001	1	<0.001	<0.001

Quality assessment of three readers for intrinsic structures on a 7-point rating scale. Mean ratings and their range are reported as well as the combined *p*-values of the Friedman-test and the *p*-values of the post hoc analysis. *p*-values for significant superiority of the PCD-CT as well as non-significant differences compared to the FPD-CT are highlighted in bold. *CT* Computed tomography, *EID-CT* Energy-integrating detector CT, *FPD-CT* Flat-panel detector CT, *PCD-CT* Photon-counting detector CT.

[1] Added data for a better overview. The FPD-CT did not provide different dose protocols or a tin filter.

Table S4 Detailed quality assessment and analysis of trabecular structures

			**Trabecular structures rating value (mean [min−max])**			***p*-values**			
Kernel	Filter	Dose protocol	PCD-CT	EID-CT	FPD-CT[1]	Combined	PCD vs. EID	PCD vs. FPD	EID vs. FPD
Br76/Ur77/normal	/	1.5 mGy	4.33 (3-6)	2.53 (1-4)	4.5 (3-5)	<0.001	**<0.001**	**1**	<0.001
		3 mGy	4.93 (4-6)	3.33 (2-5)	4.5 (3-5)	<0.001	**<0.001**	**0.403**	0.001
		6 mGy	5.93 (4-7)	3.97 (3-5)	4.5 (3-5)	<0.001	**<0.001**	**<0.001**	0.199
	Sn	1.5 mGy	4.33 (3-6)	2.3 (2-3)	4.5 (3-5)	<0.001	**<0.001**	**1**	<0.001
		0.8/1 mGy	3.19 (2-4)	1.9 (1-3)	4.5 (3-5)	<0.001	**0.001**	0.003	<0.001
		0.1/0.5 mGy	1.3 (1-2)	1.3 (1-2)	4.5 (3-5)	<0.001	1	<0.001	<0.001
Br89/Ur89/sharp	/	1.5 mGy	3.44 (2-5)	1 (1-1)	4.23 (3-6)	<0.001	**<0.001**	0.029	<0.001
		3 mGy	4.93 (2-7)	1.33 (1-3)	4.23 (3-6)	<0.001	**<0.001**	**0.307**	<0.001
		6 mGy	5.59 (3-7)	1.4 (1-3)	4.23 (3-6)	<0.001	**<0.001**	**0.013**	<0.001
	Sn	1.5 mGy	3.19 (1-4)	1 (1-1)	4.23 (3-6)	<0.001	**<0.001**	0.024	<0.001
		0.8/1 mGy	2.15 (1-3)	1 (1-1)	4.23 (3-6)	<0.001	**0.002**	<0.001	<0.001
		0.1/0.5 mGy	1 (1-1)	1 (1-1)	4.23 (3-6)	<0.001	1	<0.001	<0.001

Quality assessment of three readers for trabecular structures on a 7-point rating scale. Mean ratings and their range are reported as well as the combined *p*-values of the Friedman-test and the *p*-values of the post hoc analysis. *p*-values for significant superiority of the PCD-CT as well as non-significant differences compared to the FPD-CT are highlighted in bold. *CT* Computed tomography, *EID-CT* Energy-integrating detector CT, *FPD-CT* Flat-panel detector CT, *PCD-CT* Photon-counting detector CT.

[1] Added data for a better overview. The FPD-CT did not provide different dose protocols or a tin filter.

Table S5 Detailed quality assessment and analysis of cartilage structures

			**Cartilage structures rating value (mean [min−max])**			***p*-values**			
Kernel	Filter	Dose protocol	PCD-CT	EID-CT	FPD-CT[1]	Combined	PCD vs. EID	PCD vs. FPD	EID vs. FPD
Br76/Ur77/normal	/	1.5 mGy	4.37 (3-5)	2.2 (1-4)	5.3 (4-6)	<0.001	**<0.001**	0.043	<0.001
		3 mGy	5.07 (3-6)	2.9 (1-4)	5.3 (4-6)	<0.001	**<0.001**	**1**	<0.001
		6 mGy	5.81 (4-7)	3.6 (2-5)	5.3 (4-6)	<0.001	**<0.001**	**0.231**	<0.001
	Sn	1.5 mGy	4.26 (3-5)	2 (1-3)	5.3 (4-6)	<0.001	**<0.001**	0.013	<0.001
		0.8/1 mGy	3.37 (2-5)	1.7 (1-3)	5.3 (4-6)	<0.001	**0.001**	0.001	<0.001
		0.1/0.5 mGy	1.52 (1-2)	1.27 (1-2)	5.3 (4-6)	<0.001	1	<0.001	<0.001
Br89/Ur89/sharp	/	1.5 mGy	3.59 (2-5)	1 (1-1)	4.8 (4-6)	<0.001	**<0.001**	0.013	<0.001
		3 mGy	4.7 (3-6)	1.27 (1-2)	4.8 (4-6)	<0.001	**<0.001**	**1**	<0.001
		6 mGy	5.56 (3-7)	1.4 (1-3)	4.8 (4-6)	<0.001	**<0.001**	**0.17**	<0.001
	Sn	1.5 mGy	3.37 (2-5)	1 (1-1)	4.8 (4-6)	<0.001	**<0.001**	0.003	<0.001
		0.8/1 mGy	2.37 (1-3)	1 (1-1)	4.8 (4-6)	<0.001	**0.003**	<0.001	<0.001
		0.1/0.5 mGy	1 (1-1)	1 (1-1)	4.8 (4-6)	<0.001	1	<0.001	<0.001

Quality assessment of three readers for cartilage structures on a 7-point rating scale. Mean ratings and their range are reported as well as the combined *p*-values of the Friedman-test and the *p*-values of the post hoc analysis. *p*-values for significant superiority of the PCD-CT as well as non-significant differences compared to the FPD-CT are highlighted in bold. *CT* Computed tomography, *EID-CT* Energy-integrating detector CT, *FPD-CT* Flat-panel detector CT, *PCD-CT* Photon-counting detector CT.

[1] Added data for a better overview. The FPD-CT did not provide different dose protocols or a tin filter.
